# Inhibition of GCKIII kinases STK25 and MST3 mitigates organ lipotoxicity and enhances metabolic resilience under nutritional stress

**DOI:** 10.1186/s12916-025-04359-6

**Published:** 2025-09-22

**Authors:** Emma Andersson, Xiangdong Gongye, Emmelie Cansby, Jingjing Zhang, Mara Caputo, Bernice Asiedu, Viktor Garellick, Sheri Booten, Sue Murray, Ferran Font-Gironès, Johan Ruud, Dan Emil Lind, Manoj Amrutkar, Brian W. Howell, Ingrid Wernstedt Asterholm, Margit Mahlapuu

**Affiliations:** 1 Department of Chemistry and Molecular Biology, University of Gothenburg and Sahlgrenska University Hospital, Gothenburg, Sweden; 2 Ionis Pharmaceuticals, Carlsbad, CA USA; 3 Department of Physiology, Institute of Neuroscience and Physiology, Sahlgrenska Academy, University of Gothenburg, Gothenburg, Sweden; 4 Department of Medical Biochemistry and Cell Biology, Institute of Biomedicine, University of Gothenburgand, Sahlgrenska University Hospital , Gothenburg, Sweden; 5 Department of Pathology, Oslo University Hospital Rikshospitalet, Oslo, Norway; 6 Department of Neuroscience and Physiology, State University of New York Upstate Medical University, Syracuse, NY USA

**Keywords:** Obesity, Lipotoxicity, Thermogenic capacity, Oxidative phosphorylation

## Abstract

**Background:**

Obesity has reached pandemic proportions, highlighting the urgent need for continued research to uncover the molecular mechanisms governing lipid homeostasis and ectopic fat deposition in overnutrition. Our recent translational studies demonstrated that STE20-type kinases STK25 and MST3 associate with intracellular lipid droplets and play a pivotal role in regulating the dynamic balance between fat storage and utilization. This study aimed to assess the in vivo effects of the combined inhibition of STK25 and MST3 in obese mice.

**Methods:**

We performed phenotypic characterization in three cohorts of mice fed a high-fat diet: (1) mice with genetic ablation of *Stk25*, (2) mice treated with *Mst3*-targeting antisense oligonucleotide (ASO), and (3) mice depleted of both STK25 and MST3 by injecting *Stk25*^−/−^ mice with *Mst3* ASO. Whole-body metabolic physiology and organ lipotoxicity were examined in the STK25- and/or MST3-deficient mice compared with their respective controls by using histological assessments, immunofluorescence microscopy, molecular profiling, and biochemical assays.

**Results:**

We found that the inactivation of STK25 and MST3, either individually or in combination, provided equal protection against ectopic fat accumulation and associated lipotoxic damage in the liver, kidney, and skeletal muscle of obese mice. Strikingly, high-fat diet-fed STK25/MST3-deficient mice, but not mice lacking only one kinase, displayed reduced body and fat mass gain, which was accompanied by markedly increased abundance of thermogenesis markers in the brown adipose tissue (BAT).

**Conclusions:**

Dual inhibition of STK25 and MST3 in mice mitigates obesity-triggered lipotoxic injury to metabolic tissues and elevates indicators of BAT thermogenic capacity.

**Supplementary Information:**

The online version contains supplementary material available at 10.1186/s12916-025-04359-6.

## Background

The prevalence of obesity has tripled over the past four decades, imposing an enormous burden on human health [1]. In conditions of chronic calorie surplus, the capacity of the adipose depots to store fat is exceeded, leading to the ectopic accumulation of intracellular lipid droplets within non-adipose tissue. This triggers multiorgan lipotoxicity hallmarked by oxidative and endoplasmic reticulum (ER) stress, insulin resistance, and inflammation, ultimately resulting in cell dysfunction and death [2–5]. Ectopic fat is an independent risk factor for cardiometabolic disorders and is causally implicated in metabolic dysfunction-associated steatotic liver disease (MASLD), chronic kidney disease, type 2 diabetes, cardiovascular diseases such as hypertension, atherosclerosis, and heart failure, and several types of cancer [6–10]. Importantly, despite the high clinical relevance, the molecular mechanisms governing fat storage and utilization, as well as the biogenesis and degradation of lipid droplets, remain incompletely understood.

Our recent research has identified the STE20-type kinases comprising the germinal center kinase III (GCKIII) subfamily—STK25 (also known as YSK1 or SOK1), MST3 (also known as STK24), and MST4 (also known as STK26 or MASK)—as lipid droplet-associated proteins which critically regulate the balance of lipid storage *vs.* utilization in the context of lipotoxicity [11]. We found a significant positive correlation between the expression of *STK25*, *MST3*, and *MST4* in human liver biopsies and the severity of MASLD assessed using MAS (i.e., MASLD activity score composed of the histological scores of hepatic steatosis, lobular inflammation, and ballooning degeneration) [12–15]. We also showed that modulating the levels of the GCKIII kinases in cultured human hepatocytes, either through silencing or overexpression, markedly suppresses or aggravates, respectively, intracellular fat accumulation and oxidative/ER stress [12–17]. Consistently, the inhibition of STK25 or MST3 activity in obese mice by genetic ablation or antisense oligonucleotide (ASO) treatment effectively ameliorates steatosis, inflammatory infiltration, nutritional fibrosis, and hepatocellular damage in the liver compared with control mice [14, 18–23]. Additionally, *Stk25*^−/−^ mice fed a high-fat diet exhibit diminished ectopic lipid deposition in extrahepatic tissues, including skeletal muscle, kidneys, and vascular walls, alongside healthier adipose tissue and improved systemic glucose tolerance and insulin sensitivity [21, 24–26]. In contrast to the in vivo inactivation of STK25 or MST3, we observed that the genetic knockout of MST4 in mice has no impact on the development of diet-induced MASLD, glucose intolerance, or insulin resistance [27]. Of note, several lines of translational evidence suggest a key role of all three GCKIII kinases in promoting the initiation and progression of numerous malignancies associated with obesity and ectopic fat, including hepatocellular carcinoma (HCC), colorectal, pancreatic, and breast cancer [23, 28–39].

In this study, we investigated the in vivo impact of the combinatorial silencing of STK25 and MST3, compared to the effects of the individual inactivation of each kinase, by characterizing the whole-body metabolic physiology and organ lipotoxicity in mice under conditions of nutritional stress. We found that the depletion of STK25 and/or MST3 in mice resulted in equal protection against high-fat diet-induced lipotoxicity in the liver, kidney, and skeletal muscle. Interestingly, we also discovered that the brown adipose tissue (BAT) from obese STK25/MST3-deficient mice, but not from mice lacking only one of these kinases, exhibited markedly elevated expression of UCP1 (mitochondrial uncoupling protein 1) and related thermogenesis markers, paralleled by lower body weight and fat mass gain.

## Methods

### Animal experiments

**Fig. 1:**
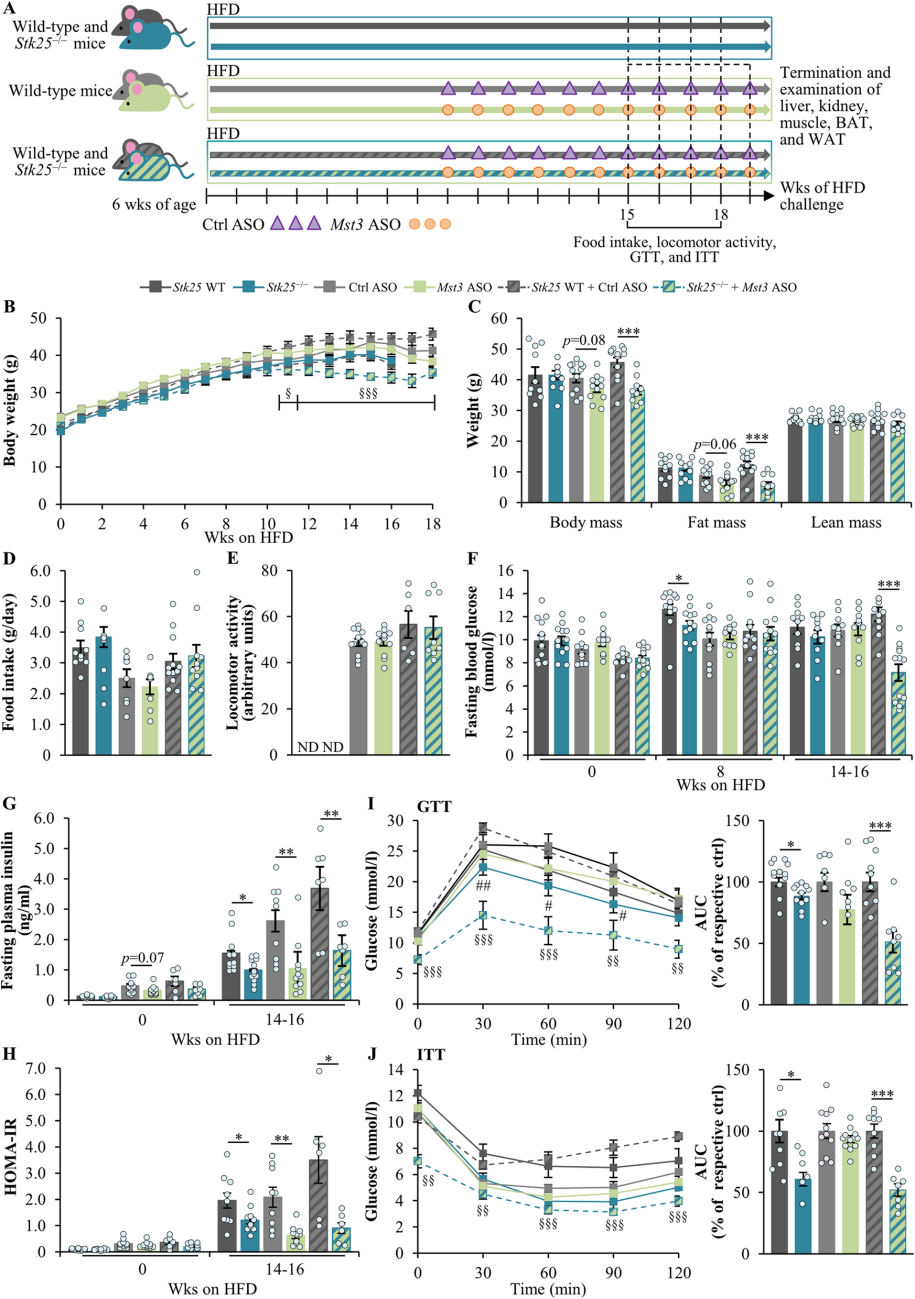
Dual inactivation of STK25 and MST3 decreases body weight and fat mass, and improves glucose and insulin homeostasis in obese mice. **A** Schematic overview of the experimental design. **B** Body weight curves. **C** Total, fat, and lean body mass measured by BCA. **D** Accumulated food consumption monitored per day. **E** Locomotor activity assessed by the open-field test. **F–G** Fasting circulating levels of glucose (**F**) and insulin (**G**). **H** HOMA-IR was calculated using the equation [fasting glucose (mg/dl) × fasting insulin (µU/ml)]/405. **I**–**J** Intraperitoneal GTT (**I**) and ITT (**J**); the area under the glucose curve in both tests is shown. Data are mean ± SEM from 6 to 13 mice per group. Values for S*tk25* knockout mice and their wild-type littermates were extracted from the previous study [21]. AUC, area under the curve; Ctrl, control; HFD, high-fat diet; ND, not determined; Wks, weeks; WT, wild-type. *p < 0.05, **p < 0.01, ***p < 0.001 *vs*. respective controls; ^§^*p* < 0.05, ^§§^*p* < 0.01, ^§§§^*p* < 0.001 for *Stk25*^–/–^ + *Mst3* ASO mice *vs*. respective controls; ^*#*^*p* < 0.05, ^*##*^*p* < 0.01 for *Stk25*^–/–^ mice *vs.* respective controls

The *Stk25* knockout mice, and mice treated with broadly acting *Mst3*-targeting ASO, were generated as previously described [21, 22]. Mice depleted of both STK25 and MST3 were created by combining genetic ablation of *Stk25* and treatment with *Mst3* ASO (see Fig. 1A for a schematic overview of the experimental design). Male mice were weaned at 3 weeks of age and housed in groups of 2–5 in a temperature-controlled (21 °C) facility with a 12-h light/dark cycle, with ad libitum access to chow diet and water. From the age of 6 weeks, mice were challenged with a pelleted high-fat diet (45 kcal% fat; D12451; Research Diets, New Brunswick, NJ) for 18–19 weeks. The body weights were recorded, blood was collected to measure glucose and insulin at different time points, and various in vivo tests were carried out as detailed below. At the age of 24–25 weeks, mice were killed by cervical dislocation under isoflurane anesthesia following a 4-h fast. Blood samples were obtained by cardiac puncture, and the liver, kidney, gastrocnemius skeletal muscle, BAT, the inguinal depot of subcutaneous white adipose tissue (sWAT), and epididymal white adipose tissue (eWAT) were harvested for histological and immunofluorescence microscopy analysis and/or snap frozen in liquid nitrogen and stored at − 80 °C for examination of protein and gene expression and biochemical assays as described below. For some of the assessments, age-matched chow-fed lean control mice of the same genetic background were included as a reference.

The mice in this study were cared for in accordance with the National Institutes of Health (NIH; Bethesda, MD) guidelines as outlined in the *Guide for the Care and Use of Laboratory Animals*. All in vivo experiments adhered to protocols approved by the local Ethics Committee for Animal Studies at the Administrative Court of Appeals in Gothenburg, Sweden (approval numbers 5.8.18–382/2011, 5.8.18–17285/2018, and 5.8.18–20691/2023).

### In vivo tests

Body composition analysis (BCA) of total, fat, and lean body mass was carried out by time-domain nuclear magnetic resonance (TD-NMR) with the Minispec LF110 Analyzer (Bruker Corporation, Rheinstetten, Germany). Food intake was measured on one occasion during the study over a 48-h period and calculated as the average consumption per 24 h. Activity was measured using an open-field test, in which mice were placed in the center of a 25 × 25 × 25 cm chamber and allowed to explore freely. Locomotor activity was then recorded over a 15-min period during the dark phase of the day in three consecutive days and analyzed using the EthoVision XT software (v8.5; Noldus, Wageningen, Netherlands). Glucose tolerance test (GTT) or insulin tolerance test (ITT) were performed after a 4-h fast by intraperitoneal injection with glucose (1 g/kg; Sigma-Aldrich, St. Louise, MO) or human recombinant insulin (1 U/kg; Actrapid Penfill; Novo Nordisk, Bagsværd, Denmark), respectively. Urine was collected during 24 h by using custom-made Perspex restraint cages.

### Cell culture and transient transfections

Mouse 3T3-L1 preadipocytes (ZenBio, Durham, NC) were cultured and differentiated into adipocytes as previously described [40]. Five days after the start of differentiation, cells were transfected with mouse *Stk25* and/or *Mst3* small interfering (si)RNA (s81845; Ambion, Austin, TX, and 169785; Invitrogen, Carlsbad, CA, respectively) or scrambled siRNA (AM4635; Invitrogen) using Lipofectamine RNAiMax (Invitrogen). Two days after transfection, cells were treated with the β3-adrenergic receptor agonist CL-316,243 (1 µM; Sigma-Aldrich) to induce browning. After an additional two days, cells were harvested for assessment of mitochondrial membrane potential and lipid content as described below.

### Histological evaluation

Tissues were fixed in 4% (vol/vol) phosphate-buffered formaldehyde (Histolab Products, Gothenburg, Sweden), embedded in paraffin, and sectioned. The liver and BAT sections were stained with hematoxylin and eosin (H&E; Histolab Products) for morphological analysis or processed for immunofluorescence or immunohistochemistry by incubating the samples with primary antibodies, followed by incubation with fluorescent dye-conjugated or biotinylated secondary antibodies (see Additional File 1: Supplementary Table S1 for antibody information). The liver sections were also stained with Picrosirius Red (Histolab Products) and counterstained with Fast Green (Sigma-Aldrich) for fibrosis investigation or dihydroethidium (DHE; Life Technologies, Grand Island, NY) to assess superoxide radical formation. MAS was analyzed in the liver sections stained with H&E according to the Kleiner/Brunt criteria adapted for rodents [41–43]. The BAT sections were also stained with MitoTracker Green (Thermo Fisher Scientific, Waltham, MA) to measure mitochondrial content. Adipocyte size distribution in the sWAT and eWAT was determined in H&E-stained tissues by a semi-automated analysis using the ImageJ software (1.47v; NIH) as described earlier [44]. Gastrocnemius skeletal muscle was embedded in optimal cutting temperature (OCT) mounting medium (Histolab Products) and frozen in liquid nitrogen, followed by cryosectioning. Cryosections were stained with Nile Red (Sigma-Aldrich) for detection of neutral lipids or subjected to enzymatic activity assays as previously described [45]. Cultured adipocytes were stained with MitoTracker Red (Thermo Fisher Scientific) or Oil Red O (Sigma-Aldrich) to examine mitochondrial membrane potential and lipid content, respectively.

The total labeled area was quantified in 6 to 8 randomly selected microscope fields (× 20; distributed over three non-consecutive tissue sections of mice) using the ImageJ software.

### Biochemical assays

The fasting blood glucose and plasma insulin levels were assessed by the Accu-Chek glucometer (Roche Diagnostics, Basel, Switzerland) and the Ultra-Sensitive Mouse Insulin ELISA Kit (Crystal Chem, Downers Grove, IL), respectively. The triacylglycerol (TAG) content was determined in the liver and kidney lysates with the Triglyceride Colorimetric Assay Kit (Cayman Chemical, Ann Arbor, MI). Glycogen concentration was measured in the liver and gastrocnemius skeletal muscle by the Glycogen Assay Kit (Sigma-Aldrich). Urine albumin and creatinine levels were evaluated by using the Mouse Albumin ELISA Kit and the Creatinine Assay Kit (both from Abcam, Cambridge, UK), respectively. The reduced and oxidized glutathione was quantified in the kidney lysates by the GSH-Glo Glutathione Assay Kit (Promega, Madison, WI). All biochemical assays were performed in duplicate.

### Western blot and RT-qPCR

Western blot was carried out as previously described (see Additional File 1: Supplementary Table S1 for antibody information) [46]. RNA was isolated using the EZNA Total RNA Kit (Omega Bio-Tek, Norcross, GA) or the RNeasy Lipid Tissue Mini Kit (Qiagen, Hilden, Germany), and cDNA was synthesized using the High-Capacity cDNA Reverse Transcription Kit (Thermo Fisher Scientific). Relative quantification was performed with the CFX Connect Real-Time System (Bio-Rad, Hercules, CA). The relative quantities of the target transcripts were normalized to the endogenous control 18S rRNA (Thermo Fisher Scientific).

### Mitochondrial copy number analysis

Mitochondrial DNA (mtDNA) copy number, an index of mitochondrial content, is quantified in eWAT samples by determining the mtDNA/nuclear DNA (nDNA) ratio using the previously validated method [47]. In brief, DNA is extracted using QIAzol (Qiagen) protocol and RT-qPCR is performed to amplify the mtDNA-encoded *mt-Nd1* and nDNA-encoded *Hk2.*

### Statistical analysis

One-way ANOVA with a two-sample Student’s *t*-test for post hoc analysis was used to evaluate statistical significance between the groups, except for body weight measurements, which were analyzed using repeated measures ANOVA with Tukey correction for multiple comparisons. Differences were considered statistically significant at *p* < 0.05. All statistical analyses were conducted using SPSS statistics (v27; IBM Corporation, Armonk, NY). All data are presented as the mean ± standard error of the mean (SEM; error bar); “*n*” signifies biological replicates, with exact sample sizes provided in the figure legends.

## Results

### Dual inhibition of STK25 and MST3 reduces weight gain and fat mass, and improves glucose tolerance and insulin sensitivity, in obese mice

Here, we compared the measures of whole-body metabolic physiology in three cohorts of high-fat diet-fed (45 kcal% fat) mice, which either lacked STK25 or MST3, or were depleted of both these kinases (see Fig. 1A for a schematic overview of the experimental design). S*tk25* knockout mice were generated by deletion of exons 4 and 5, which causes a frameshift and translational termination, as previously described [21]. To suppress MST3 abundance, the mice were treated with broadly acting *Mst3*-targeting ASO by bi-weekly intraperitoneal injections [22]. We experimentally confirmed the down-regulation of STK25 and/or MST3 protein levels in the liver, skeletal muscle, kidney, BAT, and white adipose tissue (WAT) from mice with *Stk25* knockout and/or *Mst3* knockdown using Western blot (Additional File 2: Supplementary Fig. S1). Of note, the relative expression of endogenous *STK25* and *MST3* was remarkably similar in mice across all metabolic organs studied (Additional File 2: Supplementary Fig. S2).

In agreement with our previous examinations of *Stk25*^–/–^ mice and mice treated with *Mst3* ASO [21, 22], we observed no difference in general behavior or clinical signs (body posture, mood, and motor activity) comparing the mice deficient in both STK25 and MST3 *vs*. the control group, and no local injection-site reactions were recorded. Interestingly, in contrast to *Stk25* knockout and *Mst3* knockdown mice, the total body weight and fat body mass assessed by BCA were significantly reduced by the combined silencing of STK25 and MST3 (Fig. 1B–C, Additional File 2: Supplementary Fig. S3). Notably, no change was detected in daily food intake or locomotor activity between the groups (Fig. 1D–E). Fasting blood glucose levels were lower after long-term high-fat diet challenge only in mice lacking both STK25 and MST3, while plasma insulin concentrations and HOMA of insulin resistance (HOMA-IR) values were down-regulated in all groups depleted of STK25, MST3, or STK25/MST3 compared with their respective controls (Fig. 1F–H). Intraperitoneal GTT and ITT also revealed that mice deficient in STK25 or both STK25 and MST3, but not MST3 only, displayed an improved whole-body glucose tolerance and insulin sensitivity (Fig. 1I–J), which is in line with our previous studies in *Stk25*^–/–^ and *Mst3* ASO-treated mice [21, 22]. No alterations in the peak circulating insulin concentrations (observed at 5 min after glucose administration) were found during GTT in mice depleted of STK25, MST3, or STK25/MST3, when compared with controls (data not shown).

### Inhibition of STK25 and MST3, either individually or in combination, suppresses liver steatotoxicity in high-fat diet-fed mice

**Fig. 2:**
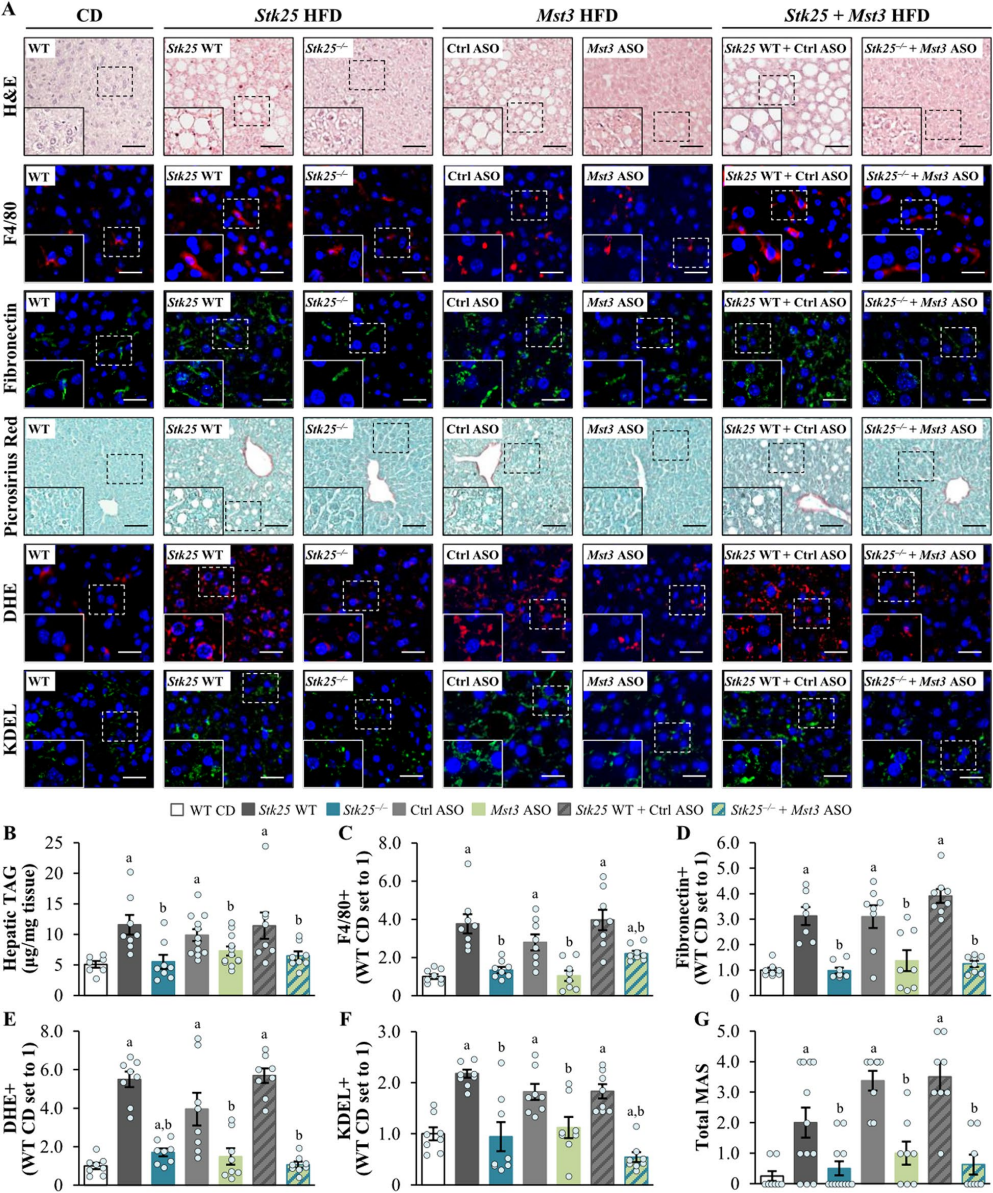
Inhibition of STK25 and/or MST3 in high-fat diet-fed mice suppresses hepatic steatotoxicity. **A** Representative images of liver sections stained with H&E, Picrosirius Red, or DHE (red), or processed for immunofluorescence with anti-F4/80 (red), anti-fibronectin (green), or anti-KDEL (green) antibodies; nuclei stained with DAPI (blue; fluorescence images). Scale bar: 50 µm. **B** Quantification of hepatic TAG content. **C–F** Quantification of the fluorescence staining for F4/80 (**C**), fibronectin (**D**), DHE (**E**), and KDEL (**F**). **G** Assessment of MAS in H&E-stained liver sections (the values for the individual histological features of MAS are presented in Additional File 2: Supplementary Fig. S5). Data are mean ± SEM from 7 to 12 mice per group. CD, chow diet; Ctrl, control; HFD, high-fat diet; WT, wild-type. ^a^*p* < 0.05 *vs*. chow-fed control mice, ^b^*p* < 0.05 *vs*. respective controls

We have previously demonstrated that *Stk25*^–/–^ mice and mice treated with *Mst3*-targeting ASO are protected from the development of diet-induced MASLD as evidenced by decreased hepatic steatosis, lobular inflammation, and nutritional fibrosis [14, 21–23]. Here, we compared the liver phenotype of mice that lacked the expression of STK25, MST3, or both kinases after a challenge with a high-fat diet. Morphological investigation of H&E-stained liver sections and colorimetric analysis of liver homogenates revealed an equal reduction in micro- and macrovesicular steatosis and TAG content, respectively, in all groups of mice depleted of STK25, MST3, or STK25/MST3 relative to their respective controls, while no difference was observed in hepatic glycogen levels (Fig. 2A–B, Additional File 2: Supplementary Fig. S4). We also found that the deficiency of STK25, MST3, or both STK25 and MST3 induced a similar suppression in liver inflammatory infiltration (measured by the abundance of Kupffer cells detected by immunostaining for F4/80) and fibrosis (assessed by immunolabeling for fibronectin and staining with Picrosirius Red detecting collagen types I and III) as opposed to the control groups (Fig. 2A, C–D). The liver sections from *Stk25* knockout and/or *Mst3* knockdown mice also displayed an equivalent reduction in the levels of oxidative stress quantified by DHE staining for superoxide radicals (O_2_^•−^) and ER stress monitored by immunostaining for KDEL (a signal motif for ER retrieval) (Fig. 2A, E–F). In line with these results, the severity of MASLD assessed by MAS (comprised of liver steatosis, lobular inflammation, and hepatocellular ballooning scores) was similarly decreased in all groups of mice depleted of either STK25, MST3, or STK25/MST3 compared with their respective controls (Fig. 2G, Additional File 2: Supplementary Fig. S5). Consistently, we observed that Mallory-Denk bodies (MDBs; visualized by immunolabeling for ubiquitin), which are known as reliable histological indices of MASLD severity and have been recently incorporated into the expanded MAS [48], were readily detected in the liver sections from the control groups of mice but not in those from mice lacking STK25 and/or MST3 (Additional File 2: Supplementary Fig. S6).

### Suppression of STK25 and/or MST3 abundance in obese mice attenuates lipotoxic injury to the kidney and skeletal muscle

**Fig. 3:**
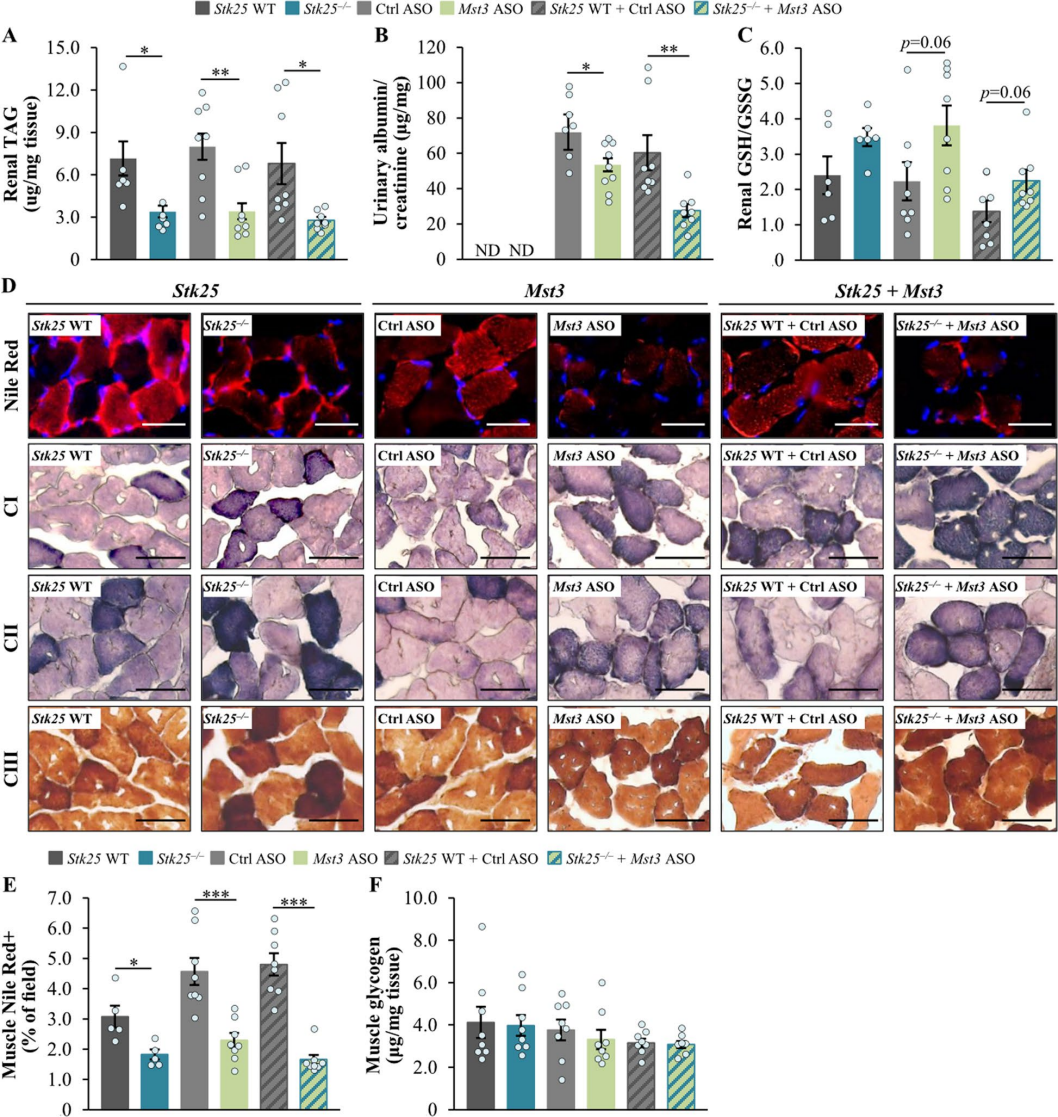
Depletion of STK25 and/or MST3 protects mice against high-fat diet-induced damage to the kidney or skeletal muscle. **A** Quantification of renal TAG content. **B** Measurement of urinary albumin to creatinine ratio (the values for each of the two parameters are presented in Additional File 2: Supplementary Fig. S7). **C** Assessment of the ratio of reduced to oxidized glutathione (GSH/GSSG) in kidney lysates. **D** Representative images of gastrocnemius skeletal muscle sections stained with Nile Red (red) or processed in enzymatic activity assays for OXPHOS complexes I, II, or III; nuclei stained with DAPI (blue; fluorescence images). Scale bars: 25 µm. **E** Quantification of Nile Red staining in skeletal muscle sections. **F** Measurement of glycogen content in skeletal muscle lysates. Data are mean ± SEM from 5 to 8 mice per group. CI-III, OXPHOS complexes I–III; Ctrl, control; ND, not determined; WT, wild-type. *p < 0.05, **p < 0.01, ***p < 0.001 *vs*. respective controls

Our previous studies have shown that *Stk25*^–/–^ mice are protected against high-fat diet-induced renal steatosis and lipotoxicity-mediated kidney pathologies [24]. Here, we analyzed renal fat accumulation and antioxidant capacity as well as albuminuria in obese mice deficient in STK25 and/or MST3 *vs*. their respective control groups. We found that the suppression of MST3 abundance caused an equivalent reduction in renal TAG content and albuminuria (assessed as the urinary albumin to creatinine ratio) compared with that induced by inhibiting both STK25 and MST3 (Fig. 3A–B; Additional File 2: Supplementary Fig. S7). Furthermore, there was a similar tendency for an increased ratio of reduced to oxidized glutathione (GSH/GSSG) in the kidney lysates from mice lacking STK25 and/or MST3 (Fig. 3C).

We also discovered that the deficiency of STK25, MST3, or STK25/MST3 in obese mice was equally efficient in lowering the intramyocellular fat storage, as evidenced by decreased staining of the gastrocnemius muscle sections with the lipophilic dye Nile Red, compared with the respective controls (Fig. 3D–E). In parallel, the inhibition of STK25, MST3, or both STK25 and MST3 similarly enhanced the pigment retention in enzymatic activity assays for the mitochondrial oxidative phosphorylation (OXPHOS) complexes I (NADH dehydrogenase), II (succinate dehydrogenase), and III (cytochrome *c* oxidase) in skeletal muscle (Fig. 3D). Notably, no change was detected in total muscle glycogen content between the groups (Fig. 3F).

### Dual Inactivation of STK25 and MST3 in high-fat diet-fed mice enhances the indicators of thermogenic capacity in BAT

**Fig. 4:**
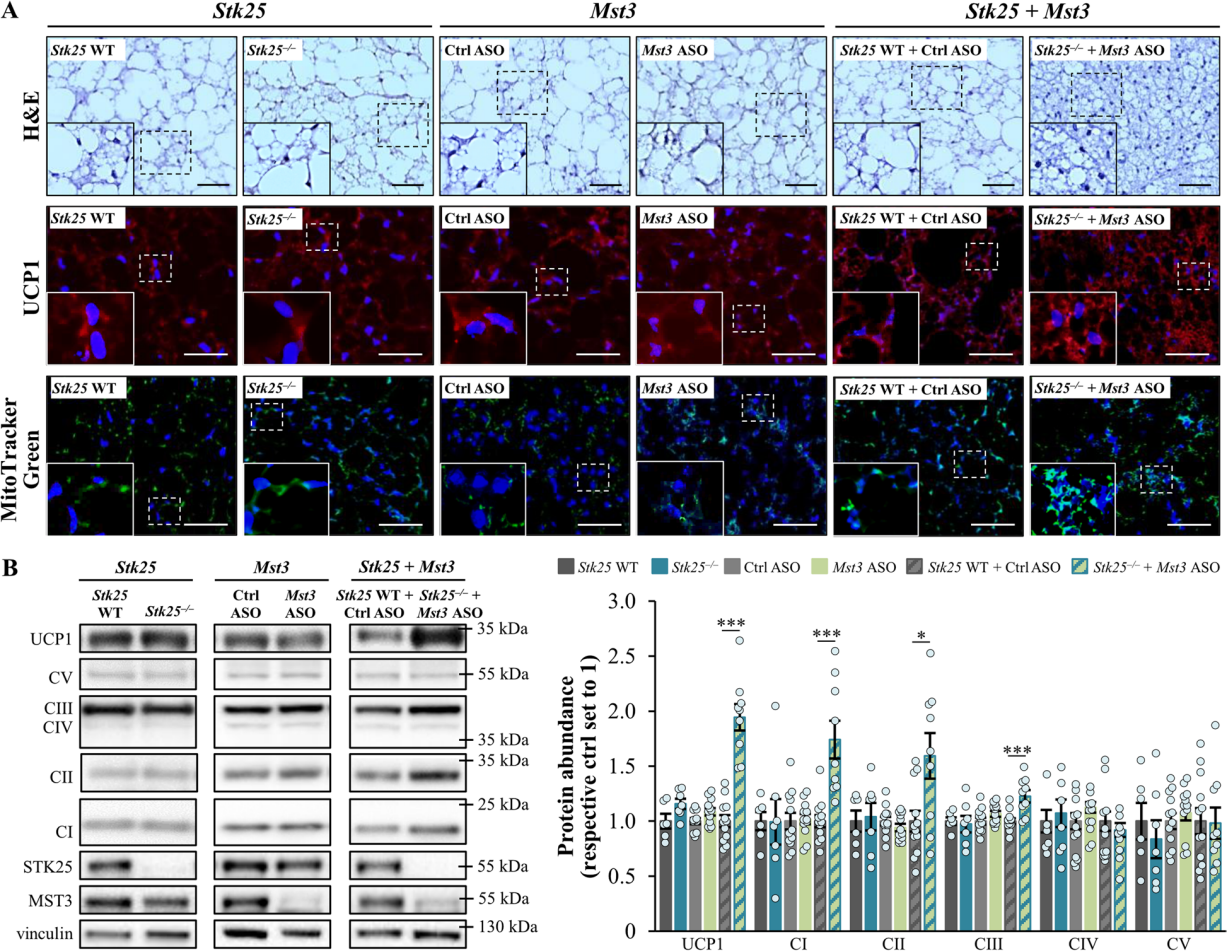
Combined silencing of STK25 and MST3 in obese mice increases the indicators of thermogenic capacity in BAT. **A** Representative images of BAT sections stained with H&E or MitoTracker Green (green), or processed for immunofluorescence with anti-UCP1 antibodies (red); nuclei stained with DAPI (blue; fluorescence images). Scale bar: 50 µm. **B** BAT lysates were analyzed by Western blot using anti‑total OXPHOS antibody cocktail or antibodies specific for UCP1, STK25, or MST3. Protein levels were quantified by densitometry; representative Western blots are shown with vinculin used as a loading control. Data are mean ± SEM from 6 to 12 mice per group. CI-V, OXPHOS complexes I–V; Ctrl, control; WT, wild-type. *p < 0.05, ***p < 0.001 *vs*. respective controls

Healthy BAT protects against high-fat diet-induced obesity and whole-body insulin resistance [40]. This prompted us to compare BAT morphology and markers of brown adipocyte activation between the groups of obese mice. Consistent with the “whitening” of BAT in obesity [49–52], we observed adipocytes with a “white-like” appearance, characterized by large lipid droplets, in the BAT sections from both high-fat diet-fed wild-type mice and mice deficient in either STK25 or MST3 (Fig. 4A). In sharp contrast, obese mice with the combined silencing of STK25 and MST3 displayed BAT with a condensed morphology and small, multilocular lipid droplets (Fig. 4A), indicating increased tissue activation and efficient utilization of fat as thermogenic fuel. In line with these results, we found that the expression of UCP1, a key regulator of thermogenesis, was approximately two-fold higher in the BAT samples from high-fat diet-fed STK25/MST3-deficient mice compared with controls (Fig. 4A–B). The abundances of OXPHOS complex I–III proteins, which are crucial for proton translocation into the mitochondrial intermembrane space required for the proton gradient driving the UCP1 activity, were also augmented in the BAT of obese mice with dual inhibition of STK25 and MST3 *vs*. the control group (Fig. 4B). These alterations were associated with increased mitochondrial content, as evidenced by enhanced MitoTracker Green staining, in the BAT sections from STK25/MST3-deficient mice (Fig. 4A). We observed no changes in UCP1 or OXPHOS proteins, or MitoTracker Green staining, in the BAT from mice lacking only one of the kinases, relative to their respective controls (Fig. 4A–B). Interestingly, the levels of tyrosine hydroxylase were comparable in the BAT samples from all groups of mice (Additional File 2: Supplementary Fig. S8), suggesting similar sympathetic nerve innervation.

**Fig. 5:**
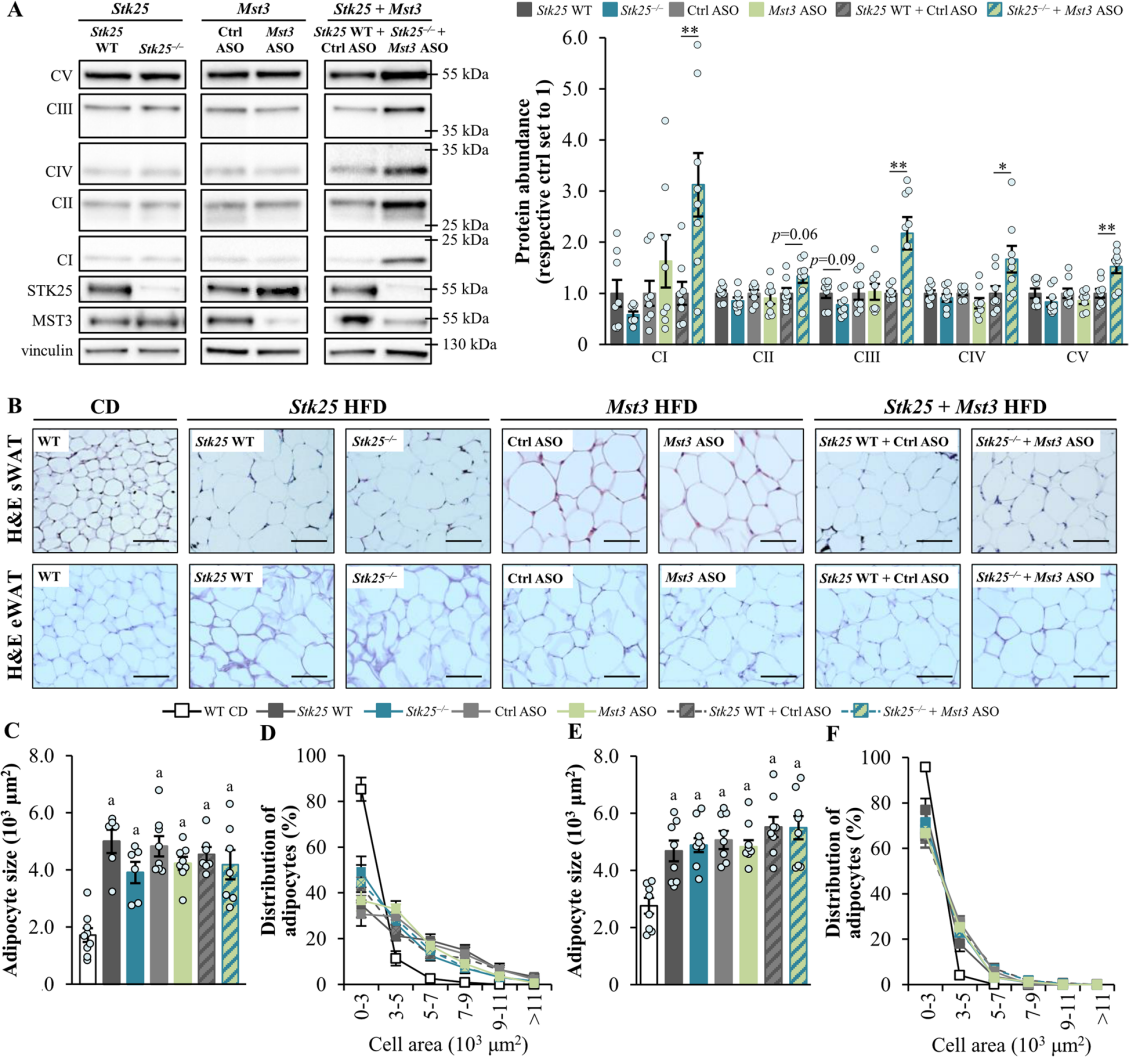
Dual inhibition of STK25 and MST3 in high-fat diet-fed mice augments oxidative phosphorylation in WAT. **A** eWAT lysates were analyzed by Western blot using anti‑total OXPHOS antibody cocktail or antibodies specific for STK25 or MST3. Protein levels were quantified by densitometry; representative Western blots are shown with vinculin used as a loading control. **B** Representative images of sWAT and eWAT sections stained with H&E. Scale bar: 100 µm. **C**–**F** The average adipocyte size and adipocyte size distribution (values representing the relative proportion of adipocytes in the given diameter class) in the sWAT (**C**,** D**) and eWAT (**E**, **F**). Data are mean ± SEM from 6 to 9 mice per group. CI-V, OXPHOS complexes I–V; CD, chow diet; Ctrl, control; HFD, high-fat diet; WT, wild-type. *p < 0.05, **p < 0.01 *vs*. respective controls; ^a^*p* < 0.05 *vs*. chow-fed control mice

The expression of OXPHOS complex I and III proteins, as well as proteins from complexes IV (cytochrome *c* oxidase) and V (ATP synthase), was increased even in the eWAT samples from obese STK25/MST3-deficient *vs*. control mice but not in mice lacking either STK25 or MST3 (Fig. 5A). Furthermore, eWAT from mice depleted of both STK25 and MST3 displayed up-regulated *Ucp1* mRNA levels and showed a trend toward higher mitochondrial content, compared to controls (Additional File 2: Supplementary Fig. S9). We did not detect any difference in the size of adipocytes in the subcutaneous or epididymal WAT depots between the groups (Fig. 5B–F).

**Fig. 6:**
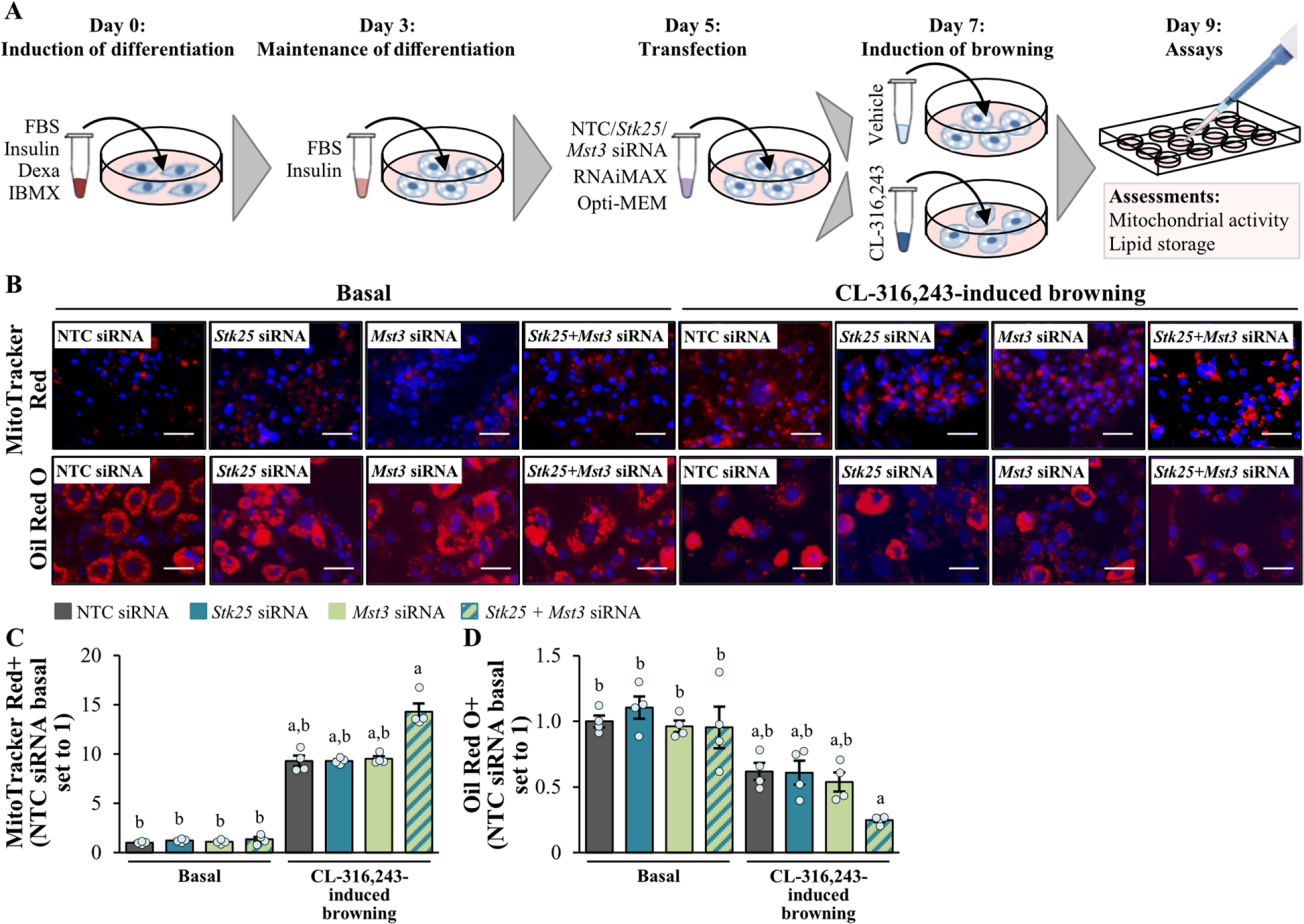
Combined silencing of STK25 and MST3 enhances mitochondrial membrane potential and reduces lipid storage in activated 3T3-L1 adipocytes. **A** Schematic overview of the experimental design. Differentiated 3T3-L1 adipocytes were transfected with *Stk25* and/or *Mst3* siRNA, or nontargeting control siRNA, and studied both under basal conditions and after browning was induced through β3-adrenergic receptor activation by CL-316,243 supplementation. **B** Representative images of cells stained with MitoTracker Red (red) or Oil Red O (red); nuclei stained with DAPI (blue). Scale bar: 25 µm. **C**–**D** Quantification of the fluorescence staining of MitoTracker Red (**C**) and Oil Red O (**D**). Data are mean ± SEM from 4 wells per group. Dexa, dexamethasone; FBS, fetal bovine serum; IBMX, 3-isobutyl-1-methylxanthine; NTC, nontargeting control. ^a^*p* < 0.05 *vs*. respective basal counterparts, ^b^*p* < 0.05 *vs*. CL-316,243-treated cells transfected with *Stk25*/*Mst3* siRNA

To test whether the changes in mitochondrial markers and lipid accumulation in the BAT of STK25/MST3-deficient mice were driven by cell-autonomous mechanism of action, we transfected 3T3-L1 adipocytes with *Stk25*- and/or *Mst3*-targeting siRNA or nontargeting control siRNA. The adipocytes were studied both under basal conditions and after browning was induced through β3-adrenergic receptor activation (see Fig. 6A for a schematic overview of the experimental design; Additional File 2: Supplementary Fig. S10). Consistent with our observation in the BAT, we found that in activated 3T3-L1 adipocytes, mitochondrial function assessed using MitoTracker Red (monitors mitochondrial membrane potential) was significantly enhanced and, conversely, fat storage analyzed by Oil Red O (stains neutral lipids) was suppressed when both STK25 and MST3 were simultaneously silenced but not when only one kinase was knocked down (Fig. 6B–D). However, we detected no differences between cells transfected with *Stk25* and/or *Mst3* siRNA or nontargeting control siRNA when cultured under basal conditions (Fig. 6B–D).

## Discussion

**Fig. 7:**
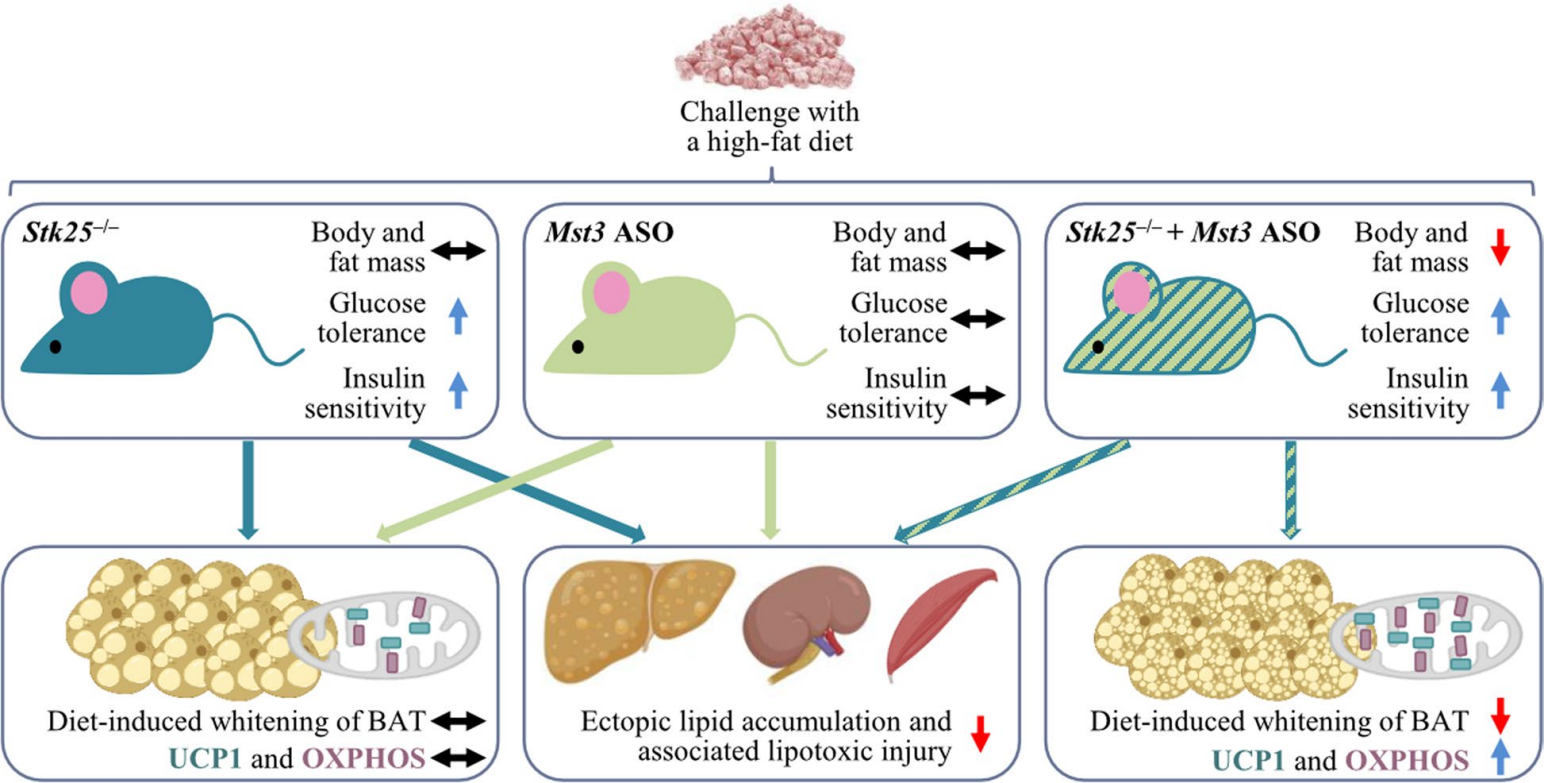
Schematic presentation illustrating the whole-body and organ-specific responses in obese mice following the inhibition of GCKIII kinases STK25 and MST3, either individually or in combination. The figure integrates findings from the current study with previous reports [14, 21, 22, 24]

The objective of this study was to examine the in vivo effects of the silencing of two closely related STE20-type kinases STK25 and MST3, either alone or in combination, using a mouse model of high-fat diet-induced obesity. First, we found that the depletion of either STK25 or MST3 caused an equal reduction in ectopic fat storage and associated lipotoxic damage in the liver, kidney, and skeletal muscle of obese mice, revealing overlapping but non-redundant roles of STK25 and MST3 in the control of lipid homeostasis (Fig. 7). Intriguingly, while the liver, kidney, and muscle of high-fat diet-fed mice lacking both STK25 and MST3 displayed changes similar to those observed when each kinase was silenced individually, the BAT from STK25/MST3-deficient mice exhibited a unique phenotype with elevated expression of thermogenesis markers, which was not seen when only one kinase was inactivated (Fig. 7), suggesting distinct interactions or a different molecular context for these proteins in brown adipocytes compared to other cell types.

Interestingly, we found that mice lacking both STK25 and MST3 gained less weight when fed a high-fat diet due to fat mass reduction, when compared with control mice. In contrast, we detected no significant difference in total body mass or fat mass in obese *Stk25* knockout or *Mst3* knockdown mice *vs*. their respective control groups, which aligns with previous research [18, 21, 22]. Of note, the combined silencing of STK25 and MST3 decreased the amount of body fat without any change in food intake or locomotor activity. Importantly, we observed that the abundance of UCP1, that uncouples mitochondrial glucose and fatty acid metabolism from ATP generation thus providing a route to dispose of excess energy, was augmented in the BAT of high-fat diet-fed STK25/MST3-deficient mice, but not in mice where only one of the kinases was inhibited. The BAT of obese mice lacking both STK25 and MST3 also contained higher levels of mitochondrial OXPHOS complex I, II, and III proteins, which have a fundamental role in UCP1-dependent respiration by driving the production of the proton motive force. Consistently, histological evaluation of the BAT from mice depleted of STK25 and MST3 revealed brown adipocytes with a condensed morphology and numerous small multilocular lipid droplets, suggesting protection against obesity-induced “whitening” of BAT. Enhanced UCP1 abundance and an increase in the thermogenic capacity of the BAT have been shown to reduce obesity and improve glucose tolerance and insulin sensitivity in high-fat diet-fed mice [53–59]. In humans, the activation of BAT as evidenced by up-regulated ^18^F-fluorodeoxyglucose uptake into the tissue and high UCP1 expression, is inversely correlated with cardiometabolic risk factors [60–65]. Notably, several pharmacological approaches promoting UCP1-mediated thermogenesis in human brown adipocytes are currently being investigated in clinical trials to mitigate obesity [60, 66]. It is therefore tempting to speculate that the lower body weight and fat mass described in obese mice depleted of both STK25 and MST3, but not in mice where only STK25 or MST3 was inhibited, may be attributed, at least in part, to elevated activity of BAT; however, direct functional assessments of thermogenesis were not performed in the current study, and this hypothesis remains to be tested. Interestingly, our in vitro experiments using 3T3-L1 adipocytes further suggested that the increase in BAT activity markers in STK25/MST3-deficient mice was derived from a cell autonomous response rather than mediated by systemic alterations.

Our previous research has revealed that *Stk25* knockout mice are protected against hepatic, renal, and skeletal muscle lipotoxicity in connection to obesity [21, 23, 24]. Similarly, the knockdown of *Mst3* in obese mice has been shown to facilitate hepatic lipid clearance and block MASLD progression [22]. Interestingly, in this study we found an equal suppression in high-fat diet-induced fat accumulation and associated lipotoxic damage in the liver, kidney, and skeletal muscle tissue when comparing mice with the combined deficiency of STK25 and MST3 *vs*. mice where only one of the kinases was depleted. These results are consistent with our recent in vitro investigations in immortalized human hepatocytes, which demonstrated that the single knockdown of STK25 or MST3 leads to a comparable decrease in intracellular lipid content and oxidative/ER stress, and no further improvement is detected when STK25 and MST3 are simultaneously silenced [17]. In conclusion, our observation of the absence of additive effects of the dual inactivation of STK25 and MST3 on hepatic, renal, and skeletal muscle lipotoxicity in obese mice suggests that, in non-adipose tissue, these kinases operate in the same signaling pathway and/or share similar mechanism(s) of action that are not amplified by combined depletion.

In agreement with our earlier studies in *Stk25*^–/–^ and *Mst3* ASO-treated mice [21, 22], we found here that the development of high-fat diet-induced hyperinsulinemia was diminished in all groups lacking STK25, MST3, or STK25/MST3. We also showed that only mice deficient in either STK25 or both STK25 and MST3 displayed increased systemic glucose tolerance and insulin sensitivity, as evidenced by improved GTT and ITT results, when challenged with a high-fat diet. In line with these findings, we have previously reported better-preserved whole-body glucose tolerance and insulin sensitivity in obese *Stk25*^–/–^ mice but not in mice dosed with the *Mst3* ASO [21, 22]. Of note, prior research has revealed that high-fat diet-fed *Mst3* gene trap mice (which exhibit a significant, though not complete, elimination of *Mst3* expression) display lower plasma insulin concentration without any alterations in body weight, fat percentage, food intake, locomotor activity, or systemic glucose tolerance [18], which is well aligned with our observations in *Mst3* ASO-treated mice. However, in contrast to mice dosed with the *Mst3* ASO, *Mst3*^–/–^ mice are also protected against the development of obesity-triggered systemic insulin resistance as demonstrated in the ITT and euglycemic–hyperinsulinemic clamp experiments [18]. The possible factors contributing to the conflicting results in insulin sensitivity in response to the pharmacological *vs.* genetic inactivation of MST3 include potential developmental effects of this kinase which would only be evident in *Mst3*^–/–^ mice, different genotypic backgrounds of mice studied (mixed 129Sv/C57BL/6J background of *Mst3* gene trap mice *vs*. pure C57BL/6J background of *Mst3* ASO-treated mice), and/or disparities in the levels of target inhibition in these two models. Importantly, since obese *Stk25*^–/–^ and *Mst3*^–/–^ mice showed improved insulin sensitivity in the absence of significant decreases in body weight and fat mass, it is likely that the protection against high-fat diet-induced insulin resistance observed in STK25/MST3-deficient mice is also mediated by metabolic adaptations beyond just reduction in body and fat mass gain.

In addition to STK25 and MST3, the GCKIII subfamily of STE20 kinases includes MST4, which shares approximately 90% amino acid sequence identity with STK25 and MST3 in the kinase domain; however, the three GCKIII kinases exhibit less than 20% similarity in the noncatalytic C-terminal domain [11]. Here, we have not attempted to inactivate MST4 in mice deficient in STK25 and/or MST3. However, compared to STK25 and MST3, the relative expression of MST4 in metabolic tissues is very low (Additional File 2: Supplementary Fig. S2), potentially indicating a minor functional relevance. Moreover, we have previously shown that the global knockout of MST4 has no impact on whole-body glucose or insulin homeostasis, adipose tissue function, or hepatic, renal, or skeletal muscle lipotoxicity in high-fat diet-fed mice [27]. Interestingly, we detected a significant decrease in Mst4 expression in both liver and BAT samples from obese STK25/MST3-deficient mice, but not in mice lacking only one of these kinases, relative to their respective controls (Additional File 2: Supplementary Fig. S11).

The present investigation has some limitations. Due to the lack of fecal sample collection, we were unable to assess whether changes in lipid excretion contributed to the metabolic alterations observed in STK25- and/or MST3-deficient mice. All in vivo experiments in this study, as well as in our previous studies performed in mice depleted of either STK25 or MST3 [14, 19–22, 24], were conducted exclusively in male mice. Potential sex-specific differences in the functions of these kinases thus remain uncharacterized, and addressing this critical knowledge gap will be the focus of our future research. Furthermore, all in vitro analyses were carried out in 3T3-L1 adipocytes examined under basal conditions and after inducing browning via β3-adrenergic receptor activation. Repeating these measurements in a brown adipocyte progenitor differentiation model, along with incorporating functional assays to monitor cellular respiration, represents an important future direction to deepen mechanistic insights into the role of GCKIII kinases in BAT.

## Conclusions

In summary, here we provide the first evidence that dual inactivation of GCKIII kinases STK25 and MST3 in mice reduces obesity-related lipotoxic damage to metabolic organs, enhances the indicators of thermogenic capacity in BAT, and improves whole-body glucose and insulin homeostasis, without raising any notable safety concerns.

## Supplementary Information

Additional file 1: Supplementary Table S1. List of antibodies

Additional file 2: Supplementary Figure S1–S11. Fig S1. Analysis of STK25 and MST3 abundance in high-fat diet-fed mice. Fig S2. Relative mRNA expression assessed by RT-qPCR in tissue samples from chow-fed mice. Fig S3. Fat mass normalized to body weight in high-fat diet-fed mice. Fig S4. Quantification of glycogen content in liver samples from high-fat diet-fed mice. Fig S5. Assessment of individual histological features of MAS in H&E-stained liver sections from high-fat diet-fed mice. Fig S6. Analysis of hepatic Mallory-Denk bodies in high-fat diet-fed mice. Fig S7. Measurement of albumin and creatinine in urine samples from high-fat diet-fed mice. Fig S8. Analysis of tyrosine hydroxylase abundance in the BAT from high-fat diet-fed mice. Fig S9. Analysis of *Ucp1* expression and mitochondrial content in eWAT samples from high-fat diet-fed mice. Fig S10. Analysis of STK25 and MST3 abundance in differentiated 3T3-L1 cells transfected with *Stk25* and/or *Mst3* siRNA, or nontargeting control siRNA, and cultured with or without CL-316,243 supplementation. Fig S11. Relative *Mst4* mRNA expression assessed by RT-qPCR in liver and BAT samples from high-fat diet-fed mice.

Additional file 3: Images of the original, uncropped Western blots

## Data Availability

The data that support the findings of this study are available on request from the corresponding author.

## Abbreviations

ASO: Antisense oligonucleotide
BAT: Brown adipose tissue
BCA: Body composition analysis
DHE: Dihydroethidium
ER: Endoplasmic reticulum
eWAT: Epididymal white adipose tissue
GCKIII: Germinal center kinase III
GSH/GSSG: Reduced and oxidized glutathione
GTT: Glucose tolerance test
H&E: Hematoxylin and eosin
HCC: Hepatocellular carcinoma
HOMA-IR: HOMA of insulin resistance
ITT: Insulin tolerance test
MAS: MASLD activity score
MASLD: Metabolic dysfunction-associated steatotic liver disease
mtDNA: Mitochondrial DNA
nDNA: Nuclear DNA
NIH: National Institutes of Health
OXPHOS: Oxidative phosphorylation
si: Small interfering
sWAT: Subcutaneous white adipose tissue
TAG: Triacylglycerol
UCP1: Uncoupling protein 1
WAT: White adipose tissue
