# Supplementary material for: Inhibition of GCKIII kinases STK25 and MST3 mitigates organ lipotoxicity and enhances metabolic resilience under nutritional stress
Source: BMC Med. 2025 Sep 22;23:518. doi: 10.1186/s12916-025-04359-6 (PMC12455811; doi:10.1186/s12916-025-04359-6)
Supplement: Supplementary file 1 — Additional file 1: Supplementary Table S1. List of antibodies [file 12916_2025_4359_MOESM1_ESM.pdf]

Supplementary Table S1. List of antibodies used for immunofluorescence/immunohistochemistry and Western blot analysis

Type	Antibody name and catalog number	Working dilution	Company
Primary antibody	anti-STK25 (25821-1-AP)	1:1000	Proteintech (Chicago, IL)
	anti-MST3 (#3723)	1:1000	Cell Signaling Technology (Boston, MA)
	anti-F4/80 (MCA497GA)	1:250	Bio-Rad (Hercules, CA)
	anti-Fibronectin (F3648)	1:300	Sigma-Aldrich (St. Louis, MO)
	anti-KDEL (ab176333)	1:500	Abcam (Cambridge, UK)
	anti-UCP1 (ab10983)	1:1000	Abcam
	anti-total OXPHOS antibody cocktail* (ab110413)	1:2000	Abcam
	anti-TH (NB300-109)	1:500	Novus Biologicals (Centennial, CO)
	anti-ubiquitin (ab7780)	1:200	Abcam
	anti-vinculin (sc-7269)	1:1000	Santa Cruz Biotechnology (Santa Cruz, CA)
Secondary antibody	Alexa Fluor-488-labeled anti-mouse IgG (A21202)	1:500	Invitrogen (Waltman, MA)
	Alexa Fluor-488-labeled anti-rabbit IgG (A11008)	1:500	Invitrogen
	Alexa Fluor-594-labeled anti-mouse IgG (A11005)	1:500	Invitrogen
	Alexa Fluor-594-labeled anti-rabbit IgG (A21207)	1:500	Invitrogen
	anti-rabbit IgG (#7074)	1:1000	Cell Signaling Technology
	anti-mouse IgG (#7076)	1:1000	Cell Signaling Technology

*This product is an optimized premixed cocktail which contains 5 mouse antibodies, one each against NDUFB8 (ab110242), SDHB (ab14714), UQCRC2 (ab14745), MTCO1 (ab14705), and ATP5A (ab14748).
