# Supplementary material for: Inhibition of GCKIII kinases STK25 and MST3 mitigates organ lipotoxicity and enhances metabolic resilience under nutritional stress
Source: BMC Med. 2025 Sep 22;23:518. doi: 10.1186/s12916-025-04359-6 (PMC12455811; doi:10.1186/s12916-025-04359-6)
Supplement: Supplementary file 2 — Additional file 2: Supplementary Figure S1–S11. Fig S1. Analysis of STK25 and MST3 abundance in high-fat diet-fed mice. Fig S2. Relative mRNA expression assessed by RT-qPCR in tissue samples from chow-fed mice. Fig S3. Fat mass normalized to body weight in high-fat diet-fed mice. Fig S4. Quantification of glycogen content in liver samples from high-fat diet-fed mice. Fig S5. Assessment of individual histological features of MAS in H&E-stained liver sections from high-fat diet-fed mice. Fig S6. Analysis of hepatic Mallory-Denk bodies in high-fat diet-fed mice. Fig S7. Measurement of albumin and creatinine in urine samples from high-fat diet-fed mice. Fig S8. Analysis of tyrosine hydroxylase abundance in the BAT from high-fat diet-fed mice. Fig S9. Analysis of Ucp1 expression and mitochondrial content in eWAT samples from high-fat diet-fed mice. Fig S10. Analysis of STK25 and MST3 abundance in differentiated 3T3-L1 cells transfected with Stk25 and/or Mst3 siRNA, or nontargeting control siRNA, and cultured with or without CL-316,243 supplementation. Fig S11. Relative Mst4 mRNA expression assessed by RT-qPCR in liver and BAT samples from high-fat diet-fed mice. [file 12916_2025_4359_MOESM2_ESM.pdf]

Supplementary Figure S1

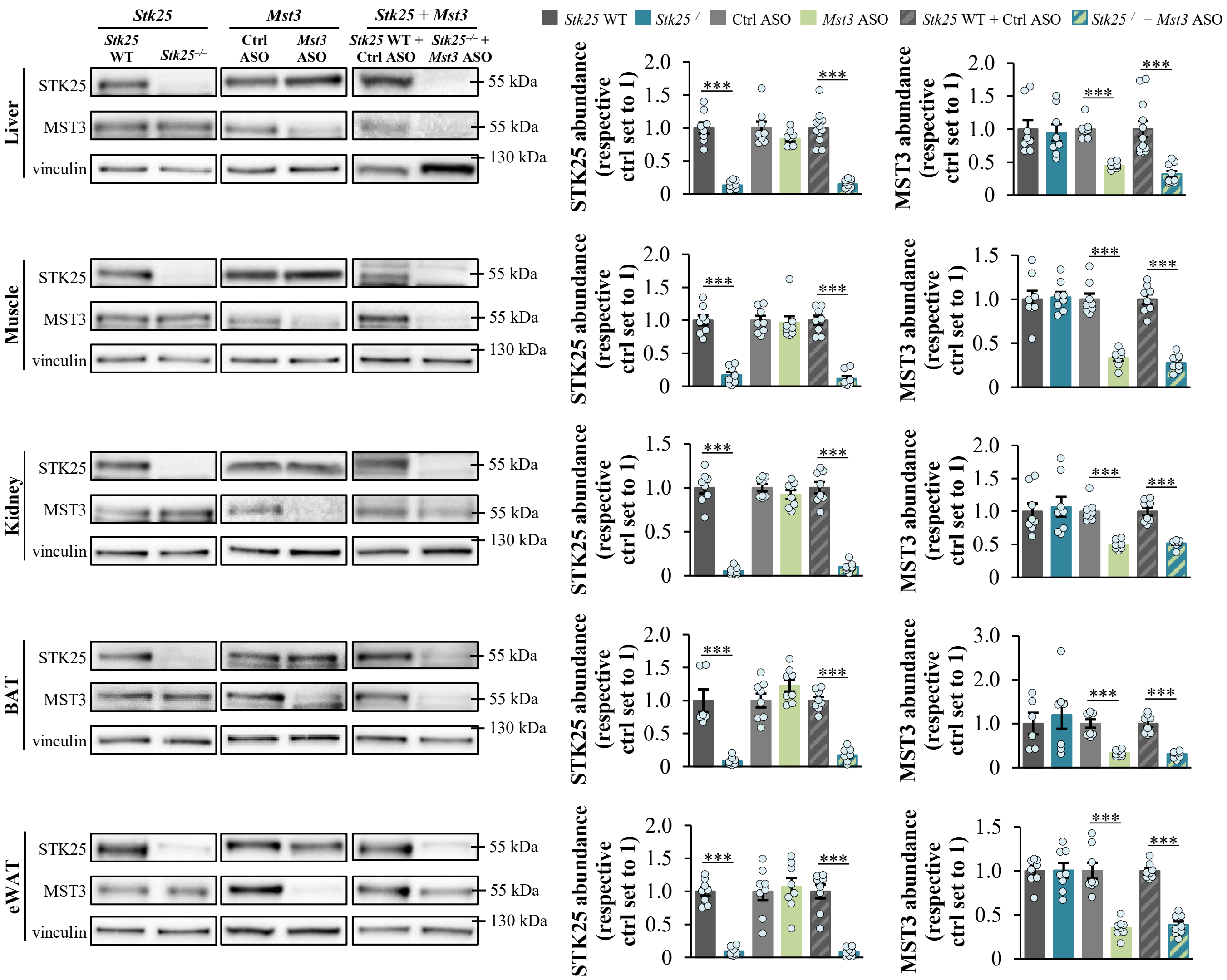

Supplementary Figure S1. Analysis of STK25 and MST3 abundance in high-fat diet-fed mice. Protein lysates from different tissues were analyzed by Western blot using antibodies specific for STK25 or MST3. Protein levels were quantified by densitometry; representative Western blots are shown with vinculin used as a loading control. Data are mean ± SEM from 6-12 mice per group. Ctrl, control; WT, wild-type. ***p<0.001 *vs.* respective controls

Supplementary Figure S2

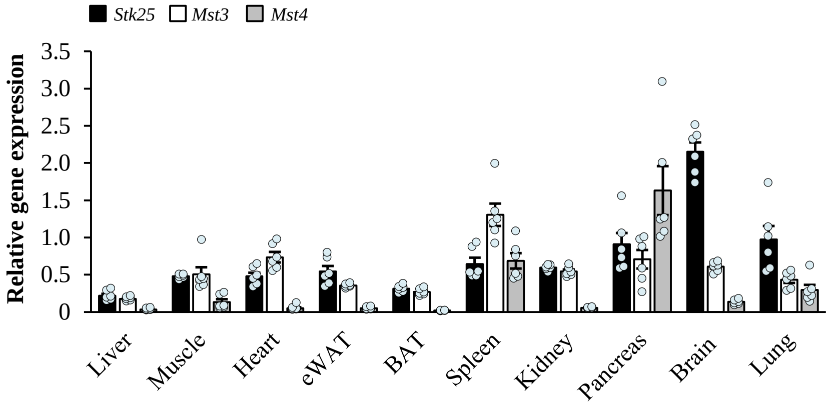

Supplementary Figure S2. Relative mRNA expression assessed by RT-qPCR in tissue samples from chow-fed mice. Data are mean ± SEM from 6 mice per group

Supplementary Figure S3

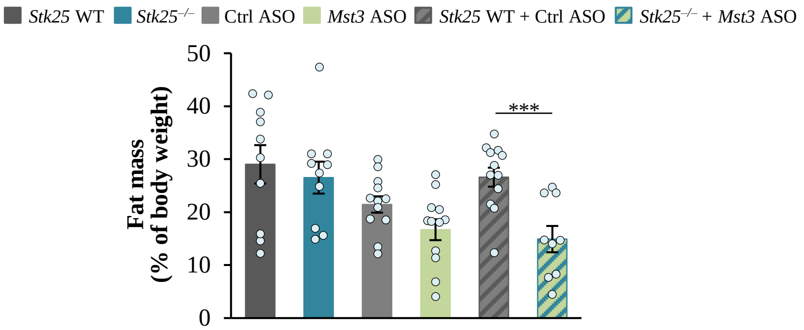

Supplementary Figure S3. Fat mass normalized to body weight in high-fat diet-fed mice. Data are mean ± SEM from 9-12 mice per group. Ctrl, control; WT, wild-type. ****p*<0.001 *vs.* respective controls

Supplementary Figure S4

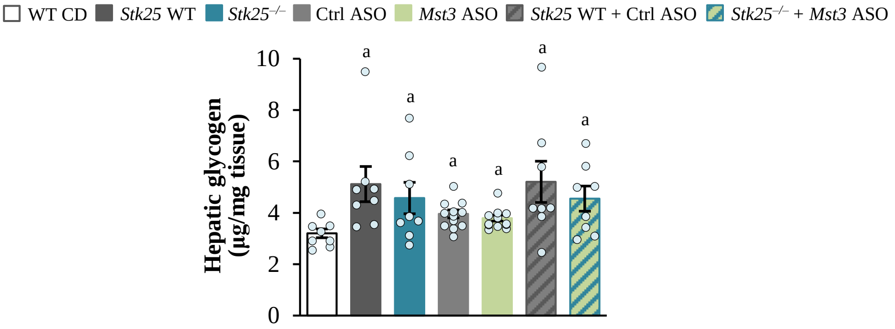

Supplementary Figure S4. Quantification of glycogen content in liver samples from high-fat diet-fed mice. Data are mean ± SEM from 8-12 mice per group. CD, chow diet; Ctrl, control; WT, wild-type. $^{a}p<0.05$ *vs.* chow-fed control mice

Supplementary Figure S5

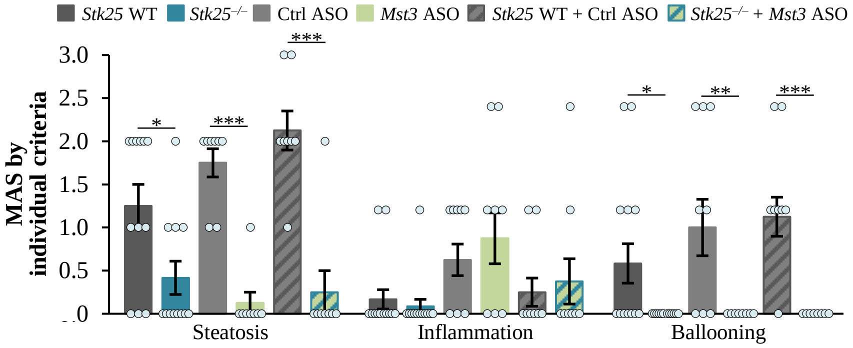

Supplementary Figure S5. Assessment of individual histological features of MAS (hepatic steatosis 0-3, lobular inflammation 0-3, hepatocellular ballooning 0-2) in H&E-stained liver sections from high-fat diet-fed mice. Data are mean ± SEM from 8-12 mice per group. Ctrl, control; WT, wild-type. *p<0.05, **p<0.01, ***p<0.001 *vs.* respective controls

Supplementary Figure S6

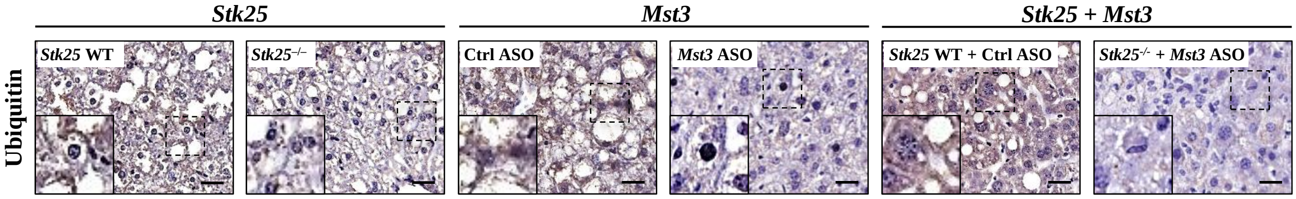

Supplementary Figure S6. Analysis of hepatic Mallory-Denk bodies in high-fat diet-fed mice. Representative images of liver sections processed for immunohistochemistry with anti-ubiquitin antibodies (brown); counterstaining with hematoxylin. Scale bar: 50 µm. Ctrl, control; WT, wild-type

Supplementary Figure S7

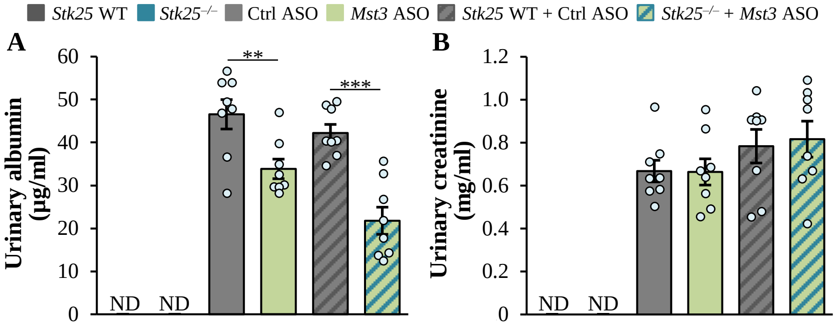

Supplementary Figure S7. Measurement of albumin (*A*) and creatinine (*B*) in urine samples from high-fat diet-fed mice. Data are mean ± SEM from 8 mice per group. Ctrl, control; ND, not determined; WT, wild-type. **$p<0.01$, ***$p<0.001$ *vs.* respective controls

Supplementary Figure S8

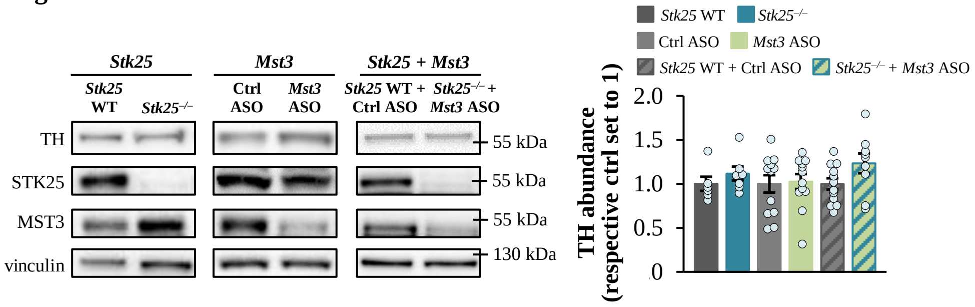

Supplementary Figure S8. Analysis of tyrosine hydroxylase abundance in the BAT from high-fat diet-fed mice. Protein lysates from BAT were analyzed by Western blot using antibodies specific for tyrosine hydroxylase, STK25, or MST3. Protein levels were quantified by densitometry; representative Western blots are shown with vinculin used as a loading control. Data are mean ± SEM from 6-12 mice per group. Ctrl, control; TH, tyrosine hydroxylase; WT, wild-type

Supplementary Figure S9

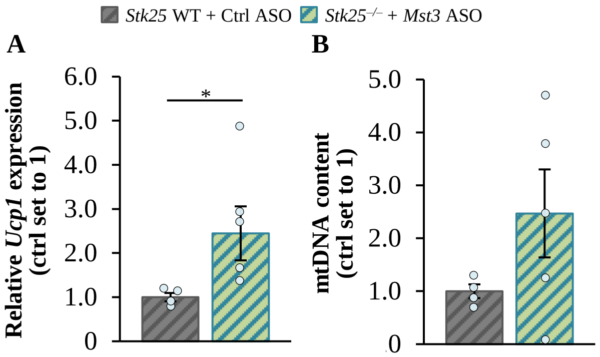

Supplementary Figure S9. Analysis of *Ucp1* expression and mitochondrial content in eWAT samples from high-fat diet-fed mice. (*A*) Relative mRNA expression of *Ucp1* was assessed by RT-qPCR. (*B*) mtDNA levels were quantified by determining the mtDNA/nDNA ratio. Data are mean ± SEM from 4-5 mice per group. Ctrl, control; WT, wild-type. *$p<0.05$ *vs.* controls

Supplementary Figure S10

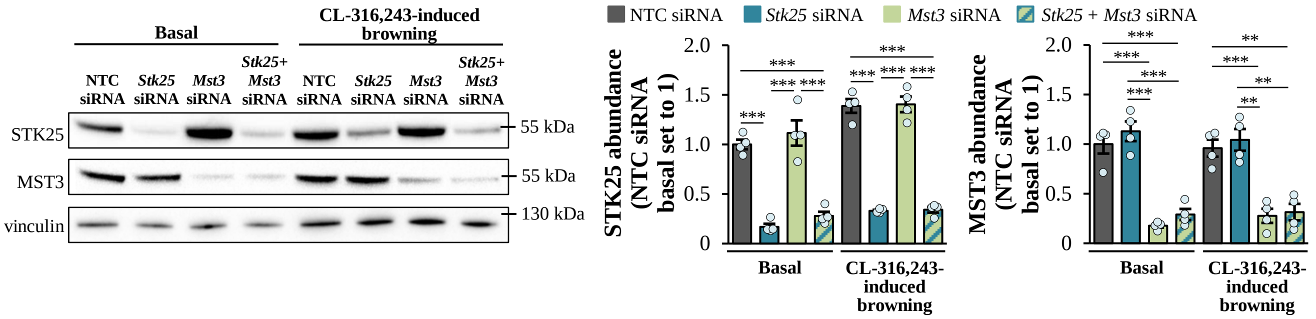

Supplementary Figure S10. Analysis of STK25 and MST3 abundance in differentiated 3T3-L1 cells transfected with *Stk25* and/or *Mst3* siRNA, or nontargeting control siRNA, and cultured with or without CL-316,243 supplementation. Protein lysates from cells were analyzed by Western blot using antibodies specific for STK25 or MST3. Protein levels were quantified by densitometry; representative Western blots are shown with vinculin used as a loading control. Data are mean ± SEM from 4 wells per group. NTC, nontargeting control. **p<0.01, ***p<0.001

Supplementary Figure S11

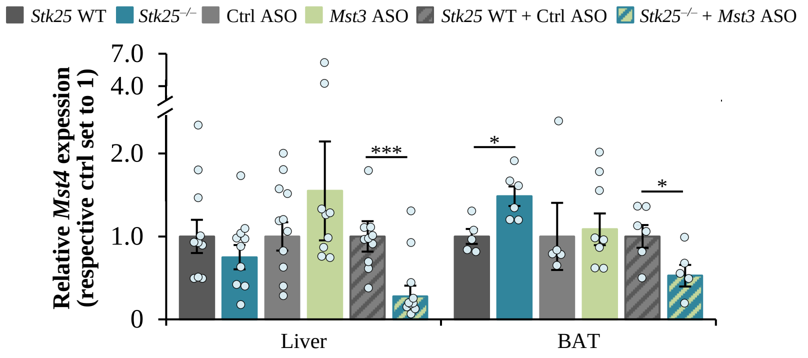

Supplementary Figure S11. Relative *Mst4* mRNA expression assessed by RT-qPCR in liver and BAT samples from high-fat diet-fed mice. Data are mean ± SEM from 5-11 mice per group. Ctrl, control; WT, wild-type. *$p<0.05$, ***$p<0.001$ *vs.* respective controls
