# Supplementary material for: Inhibition of GCKIII kinases STK25 and MST3 mitigates organ lipotoxicity and enhances metabolic resilience under nutritional stress
Source: BMC Med. 2025 Sep 22;23:518. doi: 10.1186/s12916-025-04359-6 (PMC12455811; doi:10.1186/s12916-025-04359-6)

Additional file 3. Full length uncropped original Western blots

Inhibition of GCKIII Kinases STK25 and MST3 Mitigates Organ Lipotoxicity and Enhances Metabolic Resilience Under Nutritional Stress

Emma Andersson, Xiangdong Gongye, Emmelie Cansby, Jingjing Zhang, Mara Caputo, Bernice Asiedu, Viktor Garellick, Sheri Booten, Sue Murray, Ferran Font-Gironès, Johan Ruud, Dan Emil Lind, Manoj Amrutkar, Brian W. Howell, Ingrid Wernstedt Asterholm, Margit Mahlapuu

This Additional file includes the original Western blots in Figure 4B, Figure 5A, Figure S1, Figure S8, and Figure S10. Full uncropped membranes are provided. In order to facilitate clear viewing and correspondence with representative immunoblots, all original Western blot bands in the figures of the manuscript and supporting information have been marked with red boxes.

Images of original Western blots represented in Figure 4B

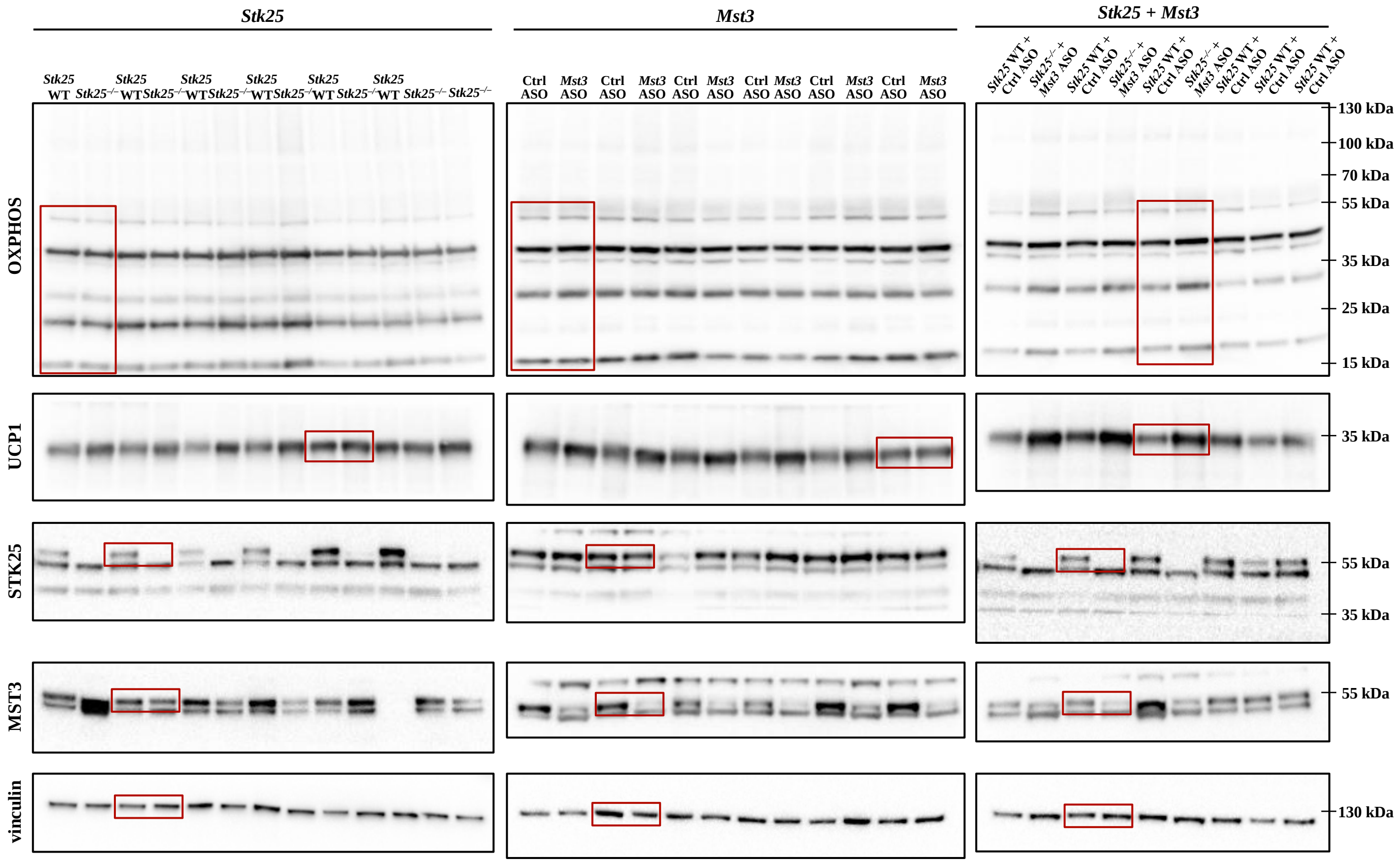

Images of original Western blots represented in Figure 5A

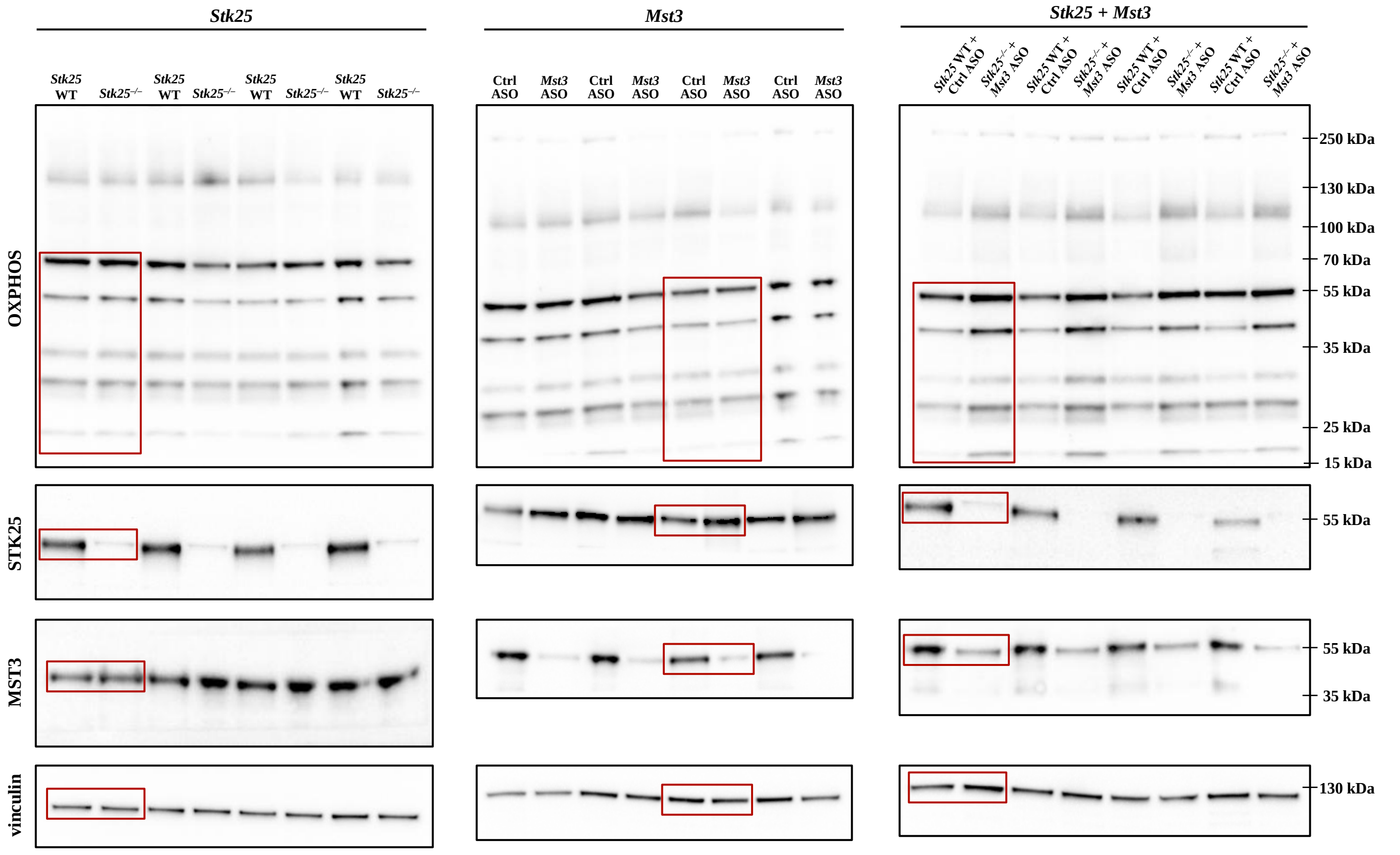

Images of original Western blots represented in Figure S1

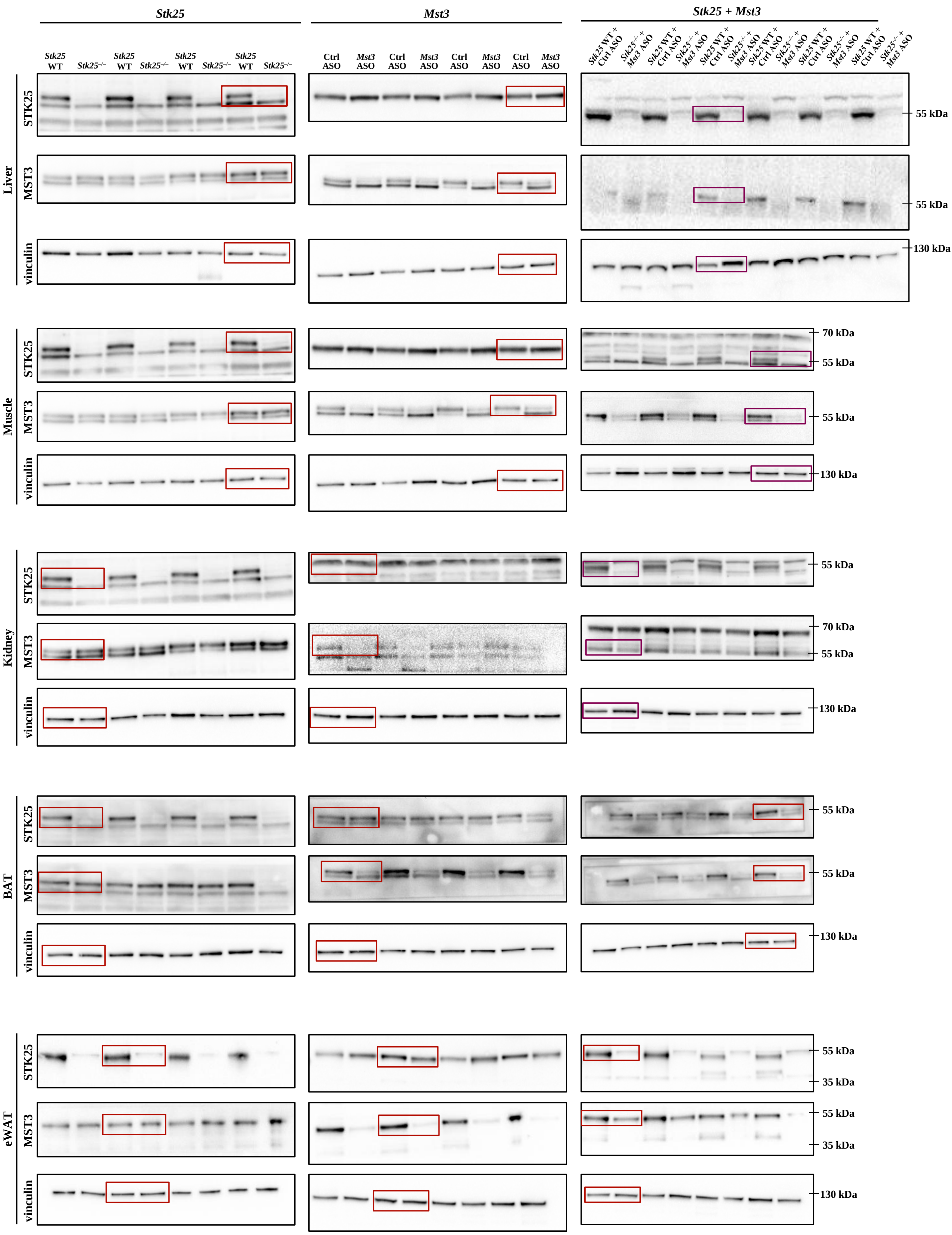

Images of original Western blots represented in Figure S8

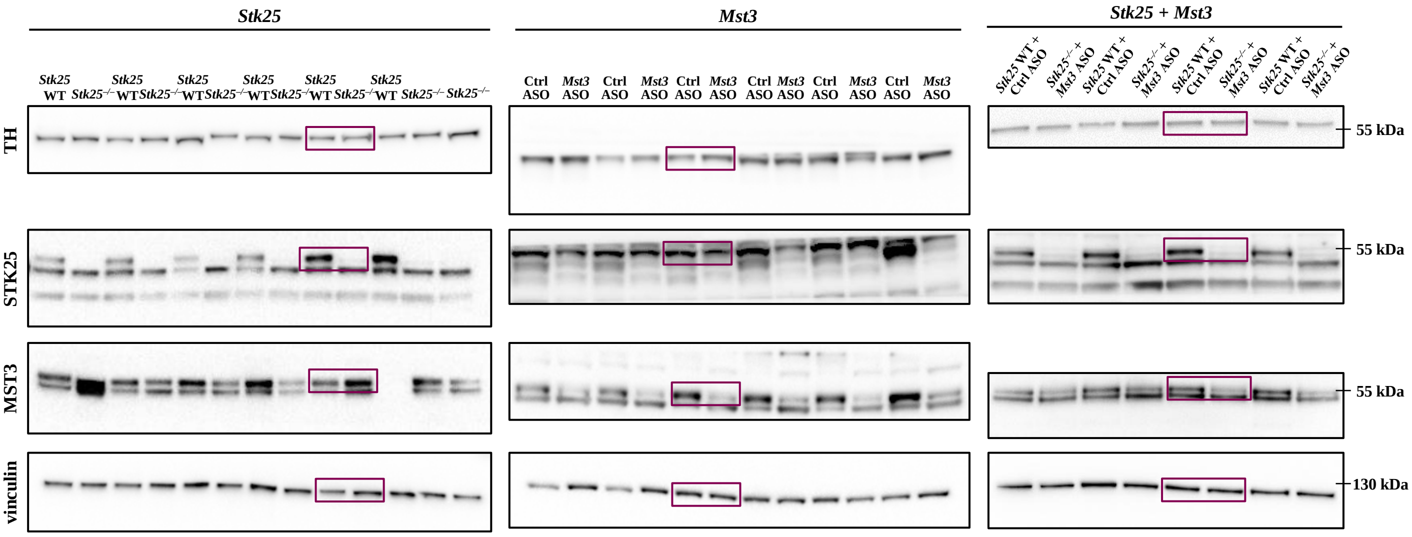

Images of original Western blots represented in Figure S10

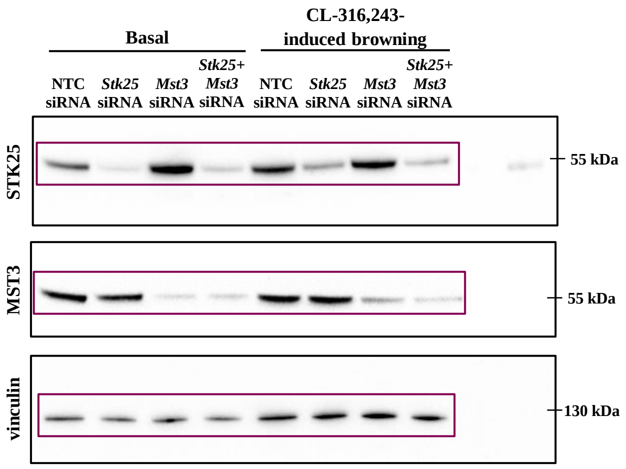

Supplement: Supplementary file 3 — Additional file 3: Images of the original, uncropped Western blots [file 12916_2025_4359_MOESM3_ESM.pdf]
